# Quantitative analysis of pyrolysis characteristics and chemical components of tobacco materials based on machine learning

**DOI:** 10.3389/fchem.2024.1353745

**Published:** 2024-02-06

**Authors:** Zhifeng Wu, Qi Zhang, Hongxiao Yu, Lili Fu, Zhen Yang, Yan Lu, Zhongya Guo, Yasen Li, Xiansheng Zhou, Yingjie Liu, Le Wang

**Affiliations:** ^1^ Zhengzhou Tobacco Research Institute of CNTC, Zhengzhou, China; ^2^ Technology Center, China Tobacco Shandong Industrial Co., Ltd., Jinan, China; ^3^ Ministry and Municipality Jointly Build the Key Laboratory of Sichuan Province for Efficient Utilization of Domestic Cigar Tobacco Leaf Industry, Chengdu, China; ^4^ Technology Center, China Tobacco Guangdong Industrial Co., Ltd., Guangzhou, China; ^5^ Qingzhou Cigarette Factory, China Tobacco Shandong Industrial Co., Ltd., Qinzhou, China

**Keywords:** tobacco material, chemical components, thermogravimetric analysis, machine learning, characteristic temperature range

## Abstract

To investigate the quantitative relationship between the pyrolysis characteristics and chemical components of tobacco materials, various machine learning methods were used to establish a quantitative analysis model of tobacco. The model relates the thermal weight loss rate to 19 chemical components, and identifies the characteristic temperature intervals of the pyrolysis process that significantly relate to the chemical components. The results showed that: 1) Among various machine learning methods, partial least squares (PLS), support vector regression (SVR) and Gaussian process regression (GPR) demonstrated superior regression performance on thermogravimetric data and chemical components. 2) The PLS model showed the best performance on fitting and prediction effects, and has good generalization ability to predict the 19 chemical components. For most components, the determination coefficients *R* ^2^ are above 0.85. While the performance of SVR and GPR models was comparable, the *R* ^2^ for most chemical components were below 0.75. 3) The significant temperature intervals for various chemical components were different, and most of the affected temperature intervals were within 130°C–400°C. The results can provide a reference for the materials selection of cigarette and reveal the possible interactions of various chemical components of tobacco materials in the pyrolysis process.

## 1 Introduction

The chemical components of tobacco raw materials affects the quality of tobacco and its products (Thielen et al., 2008). The conventional chemical components of tobacco raw materials such as total sugars, reducing sugars, nicotine, total nitrogen, potassium and chlorine are widely used in the formulation design of cigarette (Xia et al., 2009; Chen et al., 2021), quality control of raw material (Tang et al., 2020; Losso et al., 2022), and grading of raw material (Krüsemann et al., 2019; Kurt, 2021) At present, the continuous flow analyzer method is mainly used to detect the content of conventional chemical components of tobacco. However, this method is complex, time-consuming, and environmental pollution caused by the consumption of a large number of organic reagents during the detection process (Peng et al., 2022).

In view of the problems of long cycle time and poor reproducibility of conventional chemical content determination, Near Infrared Reflectance Spectroscopy (NIRS) has become an important method for the quantitative analysis of tobacco chemical components due to its simple sample preparation and fast analysis speed (Duan et al., 2012). The combination of chemical compositional measures with spectroscopic methods capable of characterizing the chemical information of a sample has been widely used in many practical applications. For example, Bi et al. (2015) combined infrared spectral data from petrol and tobacco with machine learning algorithms to show superior prediction performance for octane number in petrol and nicotine content prediction in tobacco, respectively. Zhang et al. (2008) analyzed the near-infrared spectral differences of tobacco samples, it was found that the support vector machine method can be effective for rapid and accurate analysis of conventional chemical components in tobacco. Wei et al. (2022) utilized NIRS and machine learning method to realize online monitoring of moisture, starch and other components of tobacco raw material.

The pyrolysis of tobacco, as a special type of biomass, is a very complex process due to the simultaneous presence of cellulose, hemicellulose, lignin and extractives in different ratios, resulting in multiple simultaneous reactions occurring in series parallel (Balsora et al., 2022; Guo et al., 2022). Tobacco components are degraded by different mechanisms and pathways in different temperature ranges. Pyrolysis as a thermochemical conversion process can be used to extract valuable chemicals from tobacco biomass. In addition to the NIR method discussed earlier, Thermogravimetry Analysis (TGA) is also widely used in classification, sensory quality evaluation and aroma type judgment of tobacco raw material (Guo et al., 2019; Danieli et al., 2022; Heng et al., 2022). In addition, pyrolysis data were used to predict pyrolysis products and to model kinetics in many previous studies. For example, Sun et al. (2016) developed and trained an artificial neural network (ANN) to investigate the effects of operating temperature, biomass particle size and space velocity on the pyrolysis products of pine shavings, and good agreement was achieved between the experimental and simulation results. Yin et al. (2021) classified tobacco raw materials and growth region using TGA data information sources by the SVM method, and achieved high-precision classification of tobacco raw material grade and growth region.

Based on the pyrolysis characteristics of tobacco, the biggest obstacle to building fast and accurate models for quantitative chemical composition analysis is the complex, non-linear relationship between the pyrolysis behavior of tobacco and its complex chemical composition (Strandberg et al., 2017). The potential of machine learning methods to reveal the relationship between several independent variables and several dependent variables is considerable (Jordan and Mitchell, 2015; Dobbelaere et al., 2021). Machine learning methods have been shown to be powerful in dealing with non-linear problems, for example, by using Partial Least Squares (PLS) to model the fitted data or by putting complex relationships into black boxes to build neural network models, both of which are effective in solving non-linear problems associated with complex data. This has been demonstrated in many chemical analyses related to spectra. However, it is rarely reported the quantitative analysis of pyrolysis characteristics and chemical components of tobacco raw materials by TGA methods. Based on the above background, the aim of this study is to model the quantitative relationship between pyrolysis properties and chemical composition of tobacco using machine learning methods. Specifically, 157 tobacco samples were first analyzed chemically and thermogravimetrically, respectively. Then the DTG data and chemical composition were used as inputs to establish quantitative relationships using different machine learning models, and the preferred models were used to screen the characteristic temperature intervals.

The study has the following novelty. Firstly, it is the first to directly model the quantitative analysis between the pyrolysis behavior of tobacco and 19 types of chemical information. Second, this paper finds the best model applicable between complex thermogravimetric data and chemical information by comparing different machine learning methods. Third, the temperature intervals with high correlation between different chemicals corresponding to the pyrolysis reaction process are screened out, which can provide a basis for the possible synergistic, coupling and other interaction effects of different chemicals in the pyrolysis process.

## 2 Materials and methods

### 2.1 Materials and sample preparation

The 157 tobacco samples originated from Brazil, Zimbabwe, and 104 counties in six provinces in China’s major tobacco producing regions, including Henan, Yunnan, and Guizhou provinces. The tobacco collection years included 2017, 2018, and 2019. Tobacco can be classified into 35 grades according to the national standard GB 2635-1992.After being placed in a constant temperature and humidity chamber with a temperature of (22 ± 1) °C and relative humidity of (60 ± 2)% for 48 h to reach equilibrium, the tobacco leaf samples were pulverized by means of a high-speed grinder and screened by a 60 mesh (250 μm) sieve for further use.

A total of 157 tobacco samples were studied. The chemical information included total phytoaloids, reducing sugars, total sugars, total nitrogen, potassium, chloride, starch, dichloromethane extract, solanasol, phosphate, magnesium, calcium, polyphenols, refractory acid, total amino acids, amadori compounds, neophytadiene, and PH. Tobacco samples were treated as solution according to tobacco industry standards and then analysed directly for total phytoaloids, reducing sugars, total sugars, total nitrogen, potassium, chloride, starch, dichloromethane extract, phosphate, magnesium, calcium, refractory acids, total amino acids, amadori compounds, neophytadiene, using a flow analyser (Alliance-Futura). The content of polyphenols, solanasol were determined using a liquid chromatograph. PH values were measured by a Mettler-Toledo Seven Compact PH meter.

### 2.2 Methods

#### 2.2.1 Thermogravimetric analysis

Thermogravimetric analyses of tobacco samples were finished by using discovery thermogravimetric Analyzer produced by TA Instruments. Weighing (10.0 ± 0.5) mg tobacco powder for the thermogravimetric test, the flow rate of purge gas (nitrogen) in the reaction zone of the thermogravimetric analyzer was set at 30 mL/min, and the flow rate of protection gas (nitrogen) was set at 20 mL/min. The samples were heated up from 40°C to 105°C at a rate of 10°C/min and kept for 30 min to remove the water in the samples, then heated up to 800°C at an elevated temperature rate of 10°C/min. During the test, 120 data points were recorded per minute for each sample, and the time-dependent mass loss data in the range of 105–800°C were selected. The corresponding DTG results were then obtained by normalizing and differencing the temperature-based TGA curves.

#### 2.2.2 Machine learning methods

Partial Least Squares Regression (PLS) is a multivariate statistical analysis method that extracts the latent variables with the highest correlation to the dependent variable by reducing the dimensionality of the independent variables, and then performs regression analysis on these latent variables. Compared to Principal Component Regression (PCR), PLS combines the advantages of multivariate linear regression methods and considers the relationship between independent variables and dependent variables in the selection of latent components, making it effective in handling high-dimensional data. Based on Bayesian methods, Gaussian Process Regression (GPR) is a non-parametric regression method. GPR uses a Gaussian process as a prior distribution of the data and updates the posterior distribution based on observed data to predict the new input. Support Vector Regression (SVR) is a regression method based on Support Vector Machines, minimizing loss and maximizing margin to derive the model. SVR can perform linear regression in a multidimensional feature space and achieve non-linear regression through the use of kernel functions. Random Forest Regression (RF) is a regression algorithm based on ensemble learning that constructs multiple decision trees and averages their predictions to obtain the final result. Neural Networks (NN) estimate or approximately estimate functions by connecting a large number of neurons, which can handle high-dimensional data and adapt to non-linear or complex data relationships.

#### 2.2.3 Model evaluation index

There are various metrics for machine learning to assess the effectiveness of model fitting. Root Mean Squared Error (RMSE) and coefficient of determination (*R* ^2^) are usually used to evaluate the regression models. RMSE measures the degree of deviation between the predicted values and the actual values, where a smaller RMSE indicates a higher level of model fitting accuracy. *R* ^2^ measures the extent to which the model can explain the variability in the data, also known as the coefficient of determination. It ranges from 0 to 1. The values closer to 1 indicate an improved fit of the model to the data, and closer to 0 indicate an inferior fit.

$$RMSE=\sqrt{\frac{1}{n}\sum_{i=1}^{n}{\left(y_{i}-{\hat{y}}_{i}\right)}^{2}}$$

$$R^{2}=1-\frac{\sum_{i=1}^{n}{\left(y_{i}-{\hat{y}}_{i}\right)}^{2}}{\sum_{i=1}^{n}{\left(y_{i}-\bar{y}\right)}^{2}}$$

### 2.3 Data processing

**FIGURE 1:**
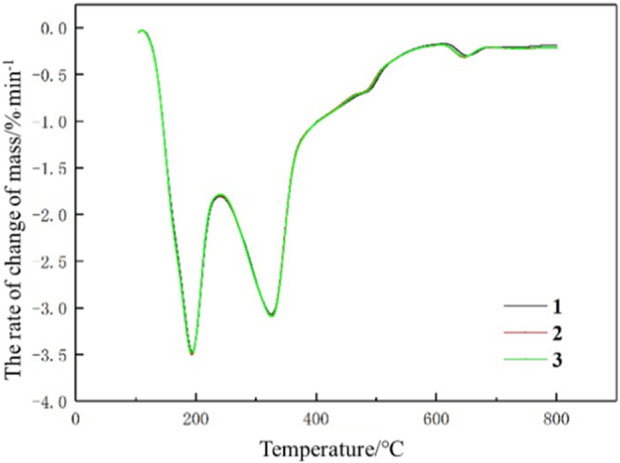
Repeatability comparison chart.

In the analysis of pyrolysis characteristic parameters of tobacco leaf samples, it is necessary to confirm the reliability of the pyrolysis characteristic data, which relies on a good repeatability of thermogravimetric experiments. Figure 1 illustrates the DTG curves of three repeated experiments carried out on a particular sample, and it can be visually observed that there are no significant differences between the three experiments. In addition, the differences between the three experiments are described quantitatively using the Normalized Root Mean Squared Error (NRMSE). NRMSE between trial 1 and trial 2 is 1.85%, and NRMSE between trial 1 and trial 3 is 0.57%. It indicates that there is a good repeatability for the thermogravimetric experiments, which is sufficient to meet the experimental requirements.

**FIGURE 2:**
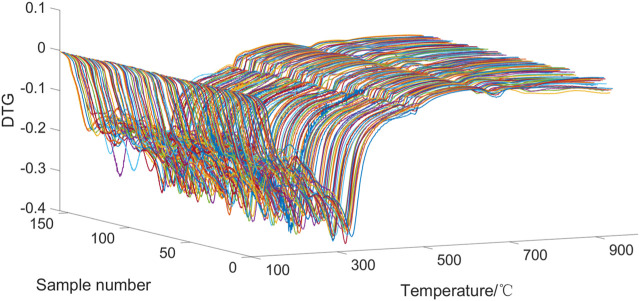
DTG curves of 157 samples.

Since the temperature intervals of the original differential thermogravimetric data (DTG) are not equal, interpolation is required to obtain thermogravimetric data from different tobacco samples at the same temperature points. The temperature range of the interpolation is from 105°C to 900°C, with a temperature interval of 0.1°C. The DTG curves of 157 samples are shown in Figure 2, with a total of 8436 points obtained for each sample. As can be seen from Figure 2, in general, the DTG curves of the 157 tobacco samples were similar in shape. The differences in the DTG curves between the tobacco samples were mainly reflected before 500°C, with less variability in the rest of the temperature range. This is because there are two distinct stages in the pyrolysis process of tobacco. The first stage is 100°C–230°C, this stage is mainly monosaccharides, free amino acids and other thermally unstable, volatile components degradation. The second stage is at 230°C–500°C, which is mainly the pyrolysis of biological components such as hemicellulose, cellulose and lignin of tobacco species (Barontini et al., 2013; Ma et al., 2022).

**FIGURE 3:**
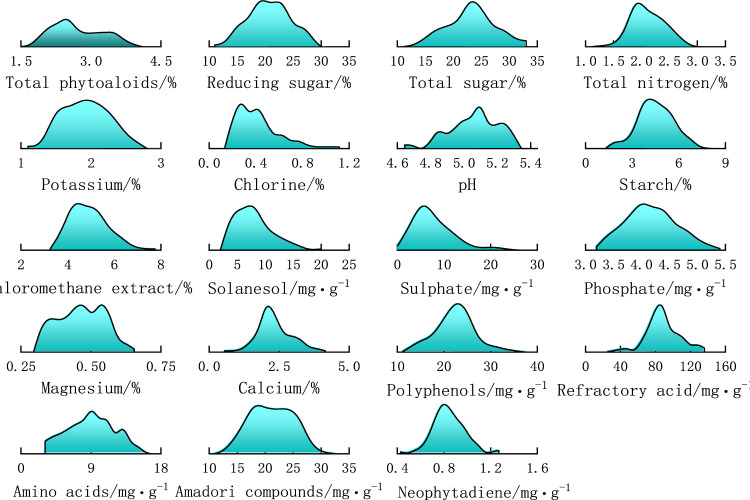
Density distribution of 19 chemical components in 157 samples.

For each chemical index, the maximum and minimum values were counted, then the distribution range was divided into a number of intervals, the number of samples in each interval was counted, and the distribution probability was calculated to make a densities plot of the distribution of the chemical content of tobacco samples. Figure 3 shows the distribution densities of 19 chemical constituents of 157 tobacco samples. The horizontal axis represents the interval of content values, and the vertical axis represents the probability density distribution. It can be seen from the figure that the distributions are not uniform for various chemical constituents.

**FIGURE 4:**
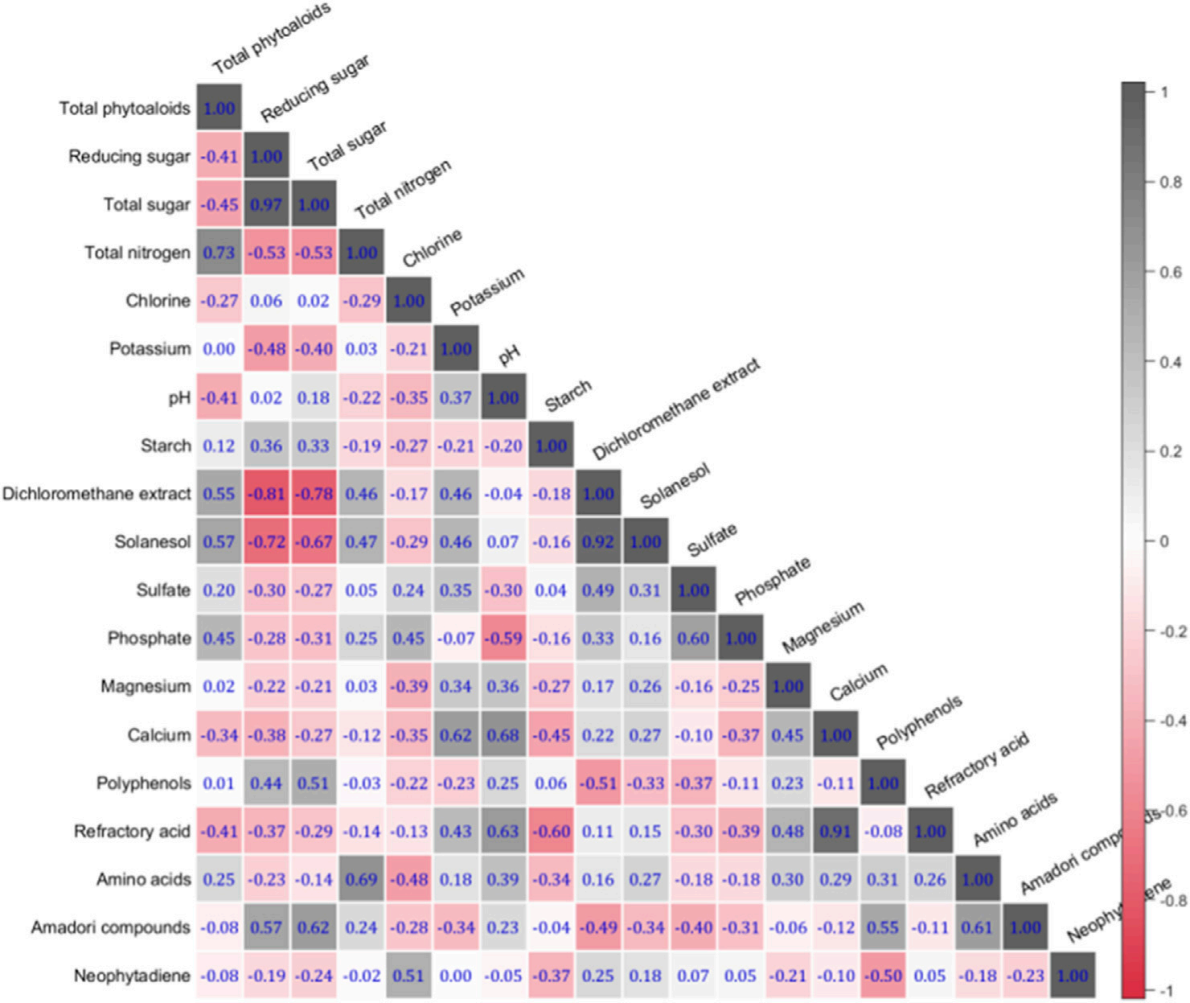
Chemical components correlation coefficient matrix.

The linear correlation coefficients among 19 chemical components of 157 tobacco samples are shown in Figure 4. The values in the heat map reflect the strength of the correlation between the chemical components (the higher the value, the stronger the correlation). It can be seen that the correlation coefficients between most components are very low, and the correlation coefficients above 0.9 are only total sugar, reducing sugar, methylene chloride extract, solanesol, calcium and nonvolatile acid. These results suggest that each chemical components needs to be modelled independently due to the dependence between different chemical components is minimal. In the end, we considered all 19 chemical information as features as inputs for machine learning.

## 3 Results and discussion

### 3.1 Performance comparison of different machine learning models

To explore quantitative analysis models between DTG data and chemical components, Partial Least Squares (PLS), Support Vector Regression (SVR), Gaussian Process Regression (GPR), Multiple Linear Regression (MLS), Random Forest (RF) and Shallow Neural Network (SNN) were used to establish fitting models for the representative chemical components of tobacco materials, including total alkaloids, reducing sugars, and total nitrogen. The regression performance of each model on the training set and validation set are shown in Table 1. It can be observed that for the three chemical components, the *R* ^2^ of the MPL, RF and RNN model training sets and test sets are below 0.7, indicating poor fitting performance for these three models on the high-dimensional DTG data. Among them, the *R* ^2^ of MPL for the three chemical components are all less than zero, indicating that linear regression cannot fit DTG data effectively. On the other hand, the R2 of the training set and test set of the PLS, SVR and GPR models are all above 0.7, indicating good fitting and prediction performance relatively. Therefore, PLS, SVR and GPR models are selected to build quantitative analysis models between DTG data and 19 chemical components for comparison in the following analysis.

**TABLE 1:** Performance comparison of different models.

	Components	PLS		SVR		GPR		MPL		RF		SNN	
		*R* ^2^	RMSE	*R* ^2^	RMSE	*R* ^2^	RMSE	*R* ^2^	RMSE	*R* ^2^	RMSE	*R* ^2^	RMSE
Training set	Total phytoaloids %	0.97	0.098	0.73	0.334	0.77	0.306	−0.18	0.615	0.59	0.3625	0.46	0.414
	Reducing sugar %	0.95	0.839	0.76	1.822	0.78	1.844	−0.87	5.464	0.67	2.3032	0.31	3.324
	Total nitrogen %	0.90	0.090	0.85	0.110	0.85	0.11	−0.9	0.381	0.57	0.1817	0.30	0.231
Test set	Total phytoaloids %	0.87	0.299	0.71	0.278	0.76	0.267	−1.24	2.619	0.41	0.684	0.33	0.874
	Reducing sugar %	0.93	2.036	0.71	2.021	0.73	1.922	−3.52	8.429	0.53	2.814	0.18	4.924
	Total nitrogen %	0.88	0.136	0.81	0.129	0.80	0.132	−2.67	1.727	0.48	0.205	0.21	0.289

### 3.2 Comparison of fitting performance for different chemical components

It is crucial to select the number of latent variables in the PLS modeling process. The model will be overfitted when there are too many latent variables, while selecting too few latent variables will result in loss of sample information and insufficient model fitting. Therefore, the number of latent variables that cumulative contribution rate of each variable reached 90% was set in the experiment, and considering the root mean square error of cross-validation (RMSECV), the number of latent variables with a cumulative contribution rate of about 90% and relatively small RMSECV were selected comprehensively.

When establishing the SVR model, the cross-validation loss is used as the goal to find the optimal penalty parameters. Considering that the GPR model performance of different kernel functions may be different, the square exponential kernel (SE), exponential kernel (Exp) and rational quadratic kernel (RQ) were selected to build models respectively, and the performance average of the three models were taken as the output result of the GPR model.

The performance comparison of the PLS, SVR and GPR models is shown in Table 2. It can be observed that for the 19 components predicted, the PLS model has higher *R* ^2^ values (0.76–0.99) and most of them are above 0.85. Moreover, it has lower RMSE for most chemical components, indicating its optimal performance. For 19 chemical components, the performance of SVR and GPR models are similar, with only a few *R* ^2^ values above 0.8 and most *R* ^2^ values below 0.75, but some RMSE values are smaller than PLS. The *R* ^2^ variation between the training sets and test sets of PLS is small, indicating that the model effectively balances fitting performance and generalization ability when selecting the number of latent variables.

**TABLE 2:** Comparison results of performance parameters of each model.

Components	Model	Training set		Test set		Latent variables
		*R* ^2^	RMSE	*R* ^2^	RMSE	
Total phytoaloids %	PLS	0.97	0.098	0.87	0.299	18
	SVR	0.73	0.334	0.71	0.278	—
	GPR	0.77	0.306	0.76	0.267	—
Reducing sugar %	PLS	0.95	0.839	0.93	2.036	16
	SVR	0.76	1.822	0.71	2.021	—
	GPR	0.76	1.721	0.73	1.922	—
Total sugar %	PLS	1.00	0.328	0.88	1.743	24
	SVR	0.79	2.028	0.72	2.249	—
	GPR	0.80	1.994	0.75	1.532	—
Total nitrogen %	PLS	0.90	0.090	0.88	0.136	6
	SVR	0.85	0.110	0.81	0.129	—
	GPR	0.85	0.110	0.80	0.132	—
Potassium %	PLS	0.89	0.273	0.86	0.104	8
	SVR	0.68	0.296	0.66	0.297	—
	GPR	0.71	0.280	0.68	0.207	—
Chlorine %	PLS	1.00	0.014	0.89	0.123	18
	SVR	0.53	0.139	0.48	0.150	—
	GPR	0.55	0.136	0.47	0.150	—
PH	PLS	0.90	0.044	0.89	0.083	14
	SVR	0.67	0.079	0.66	0.077	—
	GPR	0.70	0.083	0.68	0.059	—
Starch %	PLS	0.98	0.152	0.87	0.633	22
	SVR	0.68	0.716	0.63	0.707	—
	GPR	0.65	0.741	0.63	0.708	—
Dichloromethane extract %	PLS	0.93	0.216	0.89	0.488	25
	SVR	0.50	1.234	0.46	1.624	—
	GPR	0.47	1.492	0.43	1.788	—
Solanesol mg/g	PLS	0.97	0.606	0.90	2.106	16
	SVR	0.53	2.395	0.49	2.085	—
	GPR	0.59	2.239	0.59	1.634	—
Sulfate mg/g	PLS	0.92	1.208	0.87	2.516	12
	SVR	0.67	2.289	0.60	2.202	—
	GPR	0.79	1.815	0.78	2.057	—
Phosphate mg/g	PLS	0.90	0.275	0.84	0.245	10
	SVR	0.61	0.299	0.58	0.289	—
	GPR	0.65	0.281	0.63	0.187	—
Magnesium %	PLS	0.99	0.009	0.78	0.073	26
	SVR	0.44	0.066	0.38	0.082	—
	GPR	0.48	0.063	0.41	0.076	—
Calcium %	PLS	0.97	0.119	0.86	0.312	15
	SVR	0.48	0.402	0.43	0.494	—
	GPR	0.55	0.369	0.49	0.399	—
Polyphenols	PLS	0.80	2.634	0.76	2.554	8
	SVR	0.48	2.944	0.44	2.972	—
	GPR	0.36	3.243	0.28	2.073	—
Refractory acid	PLS	0.99	2.214	0.95	7.702	19
	SVR	0.78	9.231	0.75	7.828	—
	GPR	0.81	7.518	0.79	6.837	—
Amino acids	PLS	1.00	0.093	0.99	1.899	30
	SVR	0.75	1.707	0.72	1.925	—
	GPR	0.75	1.717	0.73	1.253	—
Amadori compounds	PLS	0.99	0.425	0.96	1.680	20
	SVR	0.81	1.645	0.75	1.864	—
	GPR	0.78	1.758	0.76	1.777	—
Neophytadiene mg/g	PLS	0.90	0.082	0.78	0.105	11
	SVR	0.42	0.101	0.33	0.089	—
	GPR	0.37	0.106	0.34	0.064	—

**FIGURE 5:**
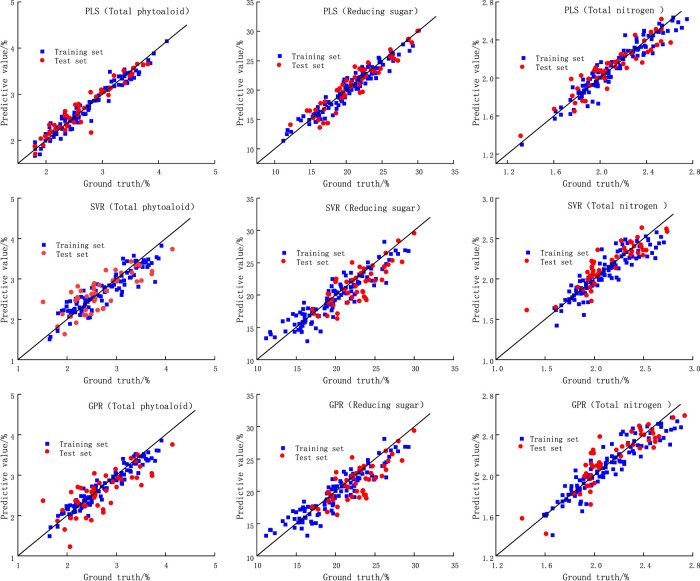
The prediction results of each model.

Figure 5 shows the fitting performance of the three models for each chemical composition in the training and test sets (only three compositions are listed, the details of the models for the other chemical compositions are presented in Table 2). Where the *x*-axis is the true value, the *y*-axis is the model prediction, and the black diagonal line is the best fit line. It can be seen that of the three constituent predictions listed, compared to the other two models, the training and test sets of the PLS model have a relatively high and high agreement between the prediction results and the ground truth, which is closer to the black diagonal line. This indicates excellent fitting and prediction performance with high accuracy. In addition, it can be seen that under the three models, the difference in *R* ^2^ and RMSE between the test set and the training set is not large, indicating that the models are not overfitted. For the PLS model in the vast majority of the above discussion shows that the PLS model used can effectively characterize the complex relationship between DTG curves and the chemical composition of tobacco materials. The PLS model with higher accuracy can be further used to investigate the relationship between the content of other components and the pyrolysis process.

### 3.3 Characteristic temperature interval analysis

PLS is a supervised multivariate statistical analysis method that combines the variability information of the dependent variable to extract latent variables. It can describe the degree and direction of the dependence of each latent variable on the original variables. The VIP (Variable importance in the projection) scores can be obtained to evaluate the contribution and explanatory ability of each variable to the model by calculating the variable coefficients, model weights and residuals of PLS regression. A higher VIP score indicates a more important variable that has a greater impact on the overall model. Given the good performance of the PLS regression model, calculating the VIP scores of the PLS regression model for the DTG of tobacco materials and the content of chemical components can help identify and select temperature points that contribute to the prediction of chemical component content significantly. This allows for the identification of temperature ranges that influence the chemical component content during pyrolysis. Generally, a VIP score greater than 1 indicates that it has important influence on the model.

**FIGURE 6:**
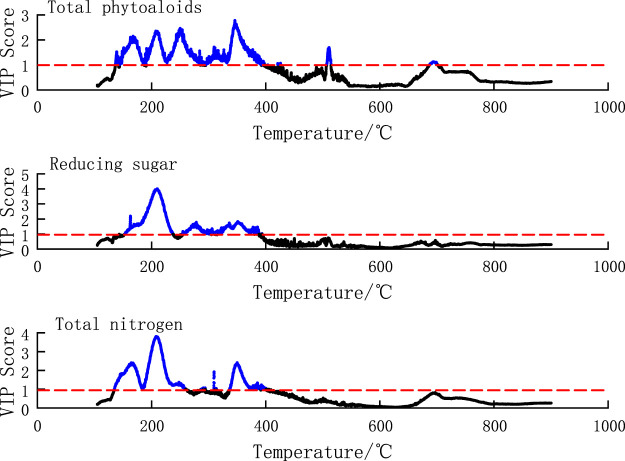
VIP score map of temperature points in components regression model.

The VIP score plot of the temperature points for chemical components is shown in Figure 6, which shows the VIP score plots for total alkaloids, reducing sugars and total nitrogen. The *x*-axis represents the temperature points, starting from 105°C. From the figure, it can be observed that the VIP scores of the temperature points for total alkaloids greater than 1 appeared within the range of 135°C–263°C and 332°C–385°C, and four distinct peaks appeared in the range of 135°C–263°C. The VIP scores of the temperature points for total nitrogen greater than 1 appeared within the range of 153°C–246°C and 260°C–399°C. The VIP scores of the temperature points for reducing sugars greater than 1 appeared within the range of 150°C–390°C, 510°C–520°C, and 688°C–701°C, and the peaks occurred near the temperature points of 135°C, 215°C, 345°C, respectively. It indicates that the pyrolysis rate in these temperature ranges has a significant impact on the regression model for the corresponding component content, especially near the peaks.

The pyrolysis temperature ranges of VIP scores greater than 1 for the 19 chemical components are shown in Table 3. It indicates that different temperature ranges have significant influences on the regression of different components of tobacco materials. The temperature ranges of VIP scores greater than 1 for chemical components are mostly within the range of 130°C–400°C, and a few of them are above 400°C. Moreover, the temperature ranges above 400°C are relatively short, indicating that the temperature ranges that have the most impact on the regression of chemical components of tobacco materials are mostly below 400°C. Combined with the samples DTG curve in Figure 2, it can be seen that the differences in the DTG curves between samples are mostly reflected within the first 400°C. This means that the DTG curves before 400°C contain the main information, and the PLS regression model effectively captures the characteristics of the DTG curves.

**TABLE 3:** 19 components correspond to temperature ranges with a VIP score greater than 1.

Chemical components	Temperature range/°C
Total phytoaloids	150-390,510-520,688-701
Reducing sugar	153-246,260-399
Total sugar	149-235,248-369
Total nitrogen	135-263,332-385
Potassium	193-281,339-382
Chlorine	135-252,238-371
pH	168-367,675-708
Starch	175-235,290-372
Dichloromethane extract	162–395
Solanesol	181-244,261-390
Sulfate	131-182,190-233,240-374,750-809
Phosphate	177-263,390-530
Magnesium	131–530
Calcium	150-275,305-373,675-710
Polyphenols	129-387,747-772
Refractory acid	150-276,300-370,375-495,683-712
Amino acids	132-234,258-295,325-370
Amadori compounds	130-229,250-342,506-512
Neophytadiene	231-284,300-374

To validate the effectiveness of the selected temperature ranges, the characteristic temperature ranges were used as independent variables to establish PLS models for total alkaloids, reducing sugars and total nitrogen. The results are shown in Table 4. Compared with the full temperature data as input, the regression performance of the models slightly decreased after the feature temperature range selection. The *R* ^2^ reduction of the training set and test set of three chemical components are within 0.1. For total alkaloids, the difference in *R* ^2^ between the test set and training set decreased after feature selection, which may be due to overfitting in the original model. For reducing sugars and total nitrogen, the difference in *R* ^2^ between the test set and training set increased after feature selection, indicating that valuable information in the independent variables was lost during the selection of the feature temperature ranges. Overall, the performance of the PLS models after feature temperature range selection did not change significantly compared with the original models, suggesting that the selected feature temperature ranges contain the main information of samples DTG curves.

**TABLE 4:** Characteristic temperature interval regression performance.

Components	Characteristic temperature range/°C	Training set				Test set			
		Before filtering *R* ^2^	After filtering *R* ^2^	Before filtering RMSE	After filtering RMSE	Before filtering *R* ^2^	After filtering *R* ^2^	Before filtering RMSE	After filtering RMSE
Total phytoaloids %	150-390,510-520,688-701	0.97	0.89	0.098	0.148	0.870	0.82	0.299	0.362
Reducing sugar %	153-246,260-399	0.95	0.90	0.839	1.373	0.93	0.81	2.036	2.997
Total nitrogen %	135-263,332-385	0.90	0.84	0.090	0.159	0.88	0.79	0.136	0.207

It is generally believed that temperature below 400°C is the main pyrolysis temperature range of monosaccharides, oligosaccharides, small organic acids, other heat-unstable, volatile components, as well as cellulose (Strandberg et al., 2017). By correlating the characteristic temperature ranges of different chemical components with the pyrolysis reaction processes of tobacco materials, it is possible to reveal the potential synergistic, coupling and catalytic effects that may exist among various components during the pyrolysis process of tobacco materials. Furthermore, the chemical component content in tobacco materials is related to sensory quality. For example, reducing sugars and total sugars are significantly positively correlated with comfort, while total alkaloids and total nitrogen are significantly negatively correlated with sensory indicators. By selecting the pyrolysis characteristic temperature ranges of different component regression models, the pyrolysis parameters within these ranges can be used as references for the selection of tobacco materials in cigarette formulation.

## 4 Conclusion

In this study, 157 kinds of tobacco materials from different growing regions, years and grades were used as samples, and the machine learning model database was constructed through experiments on the thermogravimetric analysis and chemical analysis methods of the samples. Using the differential thermogravimetric curve as the independent variable and the chemical composition content as the dependent variable, quantitative analysis and prediction models were built using different machine learning methods to predict the relationship between the heat loss rate of the differential thermogravimetric curve and the chemical composition content. The regression performance of the different machine learning models was compared, and the temperature ranges with significant effects on the chemical component content were screened based on the VIP scores of the independent variables of the best performing PLS regression model. The results show that 1) the PLS, SVR and GPR models have relatively good regression performance on DTG data and chemical component contents for the three representative chemical components tested. 2) For the prediction of 19 chemical components, the PLS model showed the best fitting, prediction and generalization ability. In addition, the *R* ^2^ values of the PLS model for most of the components were above 0.85, and the mean square errors were small. 3) The temperature range that has a large influence on most components of tobacco materials is from 130°C to 400°C, and the characteristic temperature ranges of different chemical components are different.

## Data Availability

The raw data supporting the conclusion of this article will be made available by the authors, without undue reservation.
